# *SLC11A1* (NRAMP1) Polymorphisms and Tuberculosis Susceptibility: Updated Systematic Review and Meta-Analysis

**DOI:** 10.1371/journal.pone.0015831

**Published:** 2011-01-25

**Authors:** XiangWei Li, Yu Yang, Feng Zhou, Yunzhi Zhang, Hongzhou Lu, Qi Jin, Lei Gao

**Affiliations:** 1 State Key Laboratory for Molecular Virology and Genetic Engineering, Institute of Pathogen Biology, Chinese Academy of Medical Sciences and Peking Union Medical College, Beijing, China; 2 Department of Infectious Diseases, Shanghai Public Health Clinical Center, Fudan University, Shanghai, China; Hopital Raymond Poincare - Universite Versailles St. Quentin, France

## Abstract

**Background:**

Natural resistance associated macrophage protein 1 (NRAMP1), encoded by the *SLC11A1* gene, has been described to regulate macrophage activation and be associated with infectious and autoimmune diseases. The relation between *SLC11A1* polymorphisms and tuberculosis susceptibility has been studied in different populations.

**Methods:**

We systematically reviewed published studies on *SLC11A1* polymorphisms and tuberculosis susceptibility until September 15, 2010 and quantitatively summarized associations of the most widely studied polymorphisms using meta-analysis.

**Results:**

In total, 36 eligible articles were included in this review. In Meta-analysis, significant associations were observed between tuberculosis risk and widely studied *SLC11A1* polymorphisms with summarized odds ratio of 1.35 (95%CI, 1.17–1.54), 1.25 (95% CI, 1.04–1.50), 1.23 (95% CI, 1.04–1.44), 1.31 (95%CI, 1.08–1.59) for 3′ UTR, D543N, INT4, and 5′ (GT)n, respectively. Heterogeneity between studies was not pronounced, and the associations did not remarkably vary in the stratified analysis with respect to study population and study base.

**Conclusions:**

The association between *SLC11A1* polymorphisms and tuberculosis susceptibility observed in our analyses supports the hypothesis that NRAMP1 might play an important role in the host defense to the development of tuberculosis.

## Introduction

Host genetic susceptibility to infectious disease has been widely studied in recent years, which is helpful for high-risk population identification and therefore promotes diseases prevention and early diagnosis [1], [2]. Moreover, such study also contributes to clarify potential mechanisms underlying host defense to the disease development. Natural resistance associated macrophage protein 1 (NRAMP1), encoded by the *SLC11A1* gene, has multiple effects on macrophage activation and has been reported to play an important role in host innate immune response against infections [3].

Tuberculosis (TB), caused by infection of *Mycobacterium tuberculosis*, remains a major challenge to global public health. As estimated, that one-third of the world's population is infected, but that only a minority of those infected ever develop TB [4]. Host genetic susceptibility, together with some environmental and lifestyle factors, has been suggested to contribute to such clinical diversity [5], [6]. In 1998, for the first time, relation between *SLC11A1* polymorphisms and TB susceptibility was reported in a population from West Africa [7]. Later, the association of several *SLC11A1* loci have been extensively investigated, including 3′ UTR (1729+55del4), D543N (Asp543Asn), INT4 (469+14G/C), and 5′ promoter (GT)n. However, the results were not consistent between the studies. A meta-analysis, which based on literature review until December 2004, suggested an ethnicity specific effect of *SLC11A1* polymorphisms on TB risk [8]. In the past five years, the number of original studies addressing this topic has doubled. Therefore, it is necessary to update the meta-analysis which might provide more solid evidence and minimize potential bias caused by limited publications in the past.

In this article, we performed a systematic review and meta-analysis, based on literature identification until 15 September 2010, to summarize associations between the most widely studied *SLC11A1* polymorphisms and TB susceptibility.

## Results

**Figure 1:**
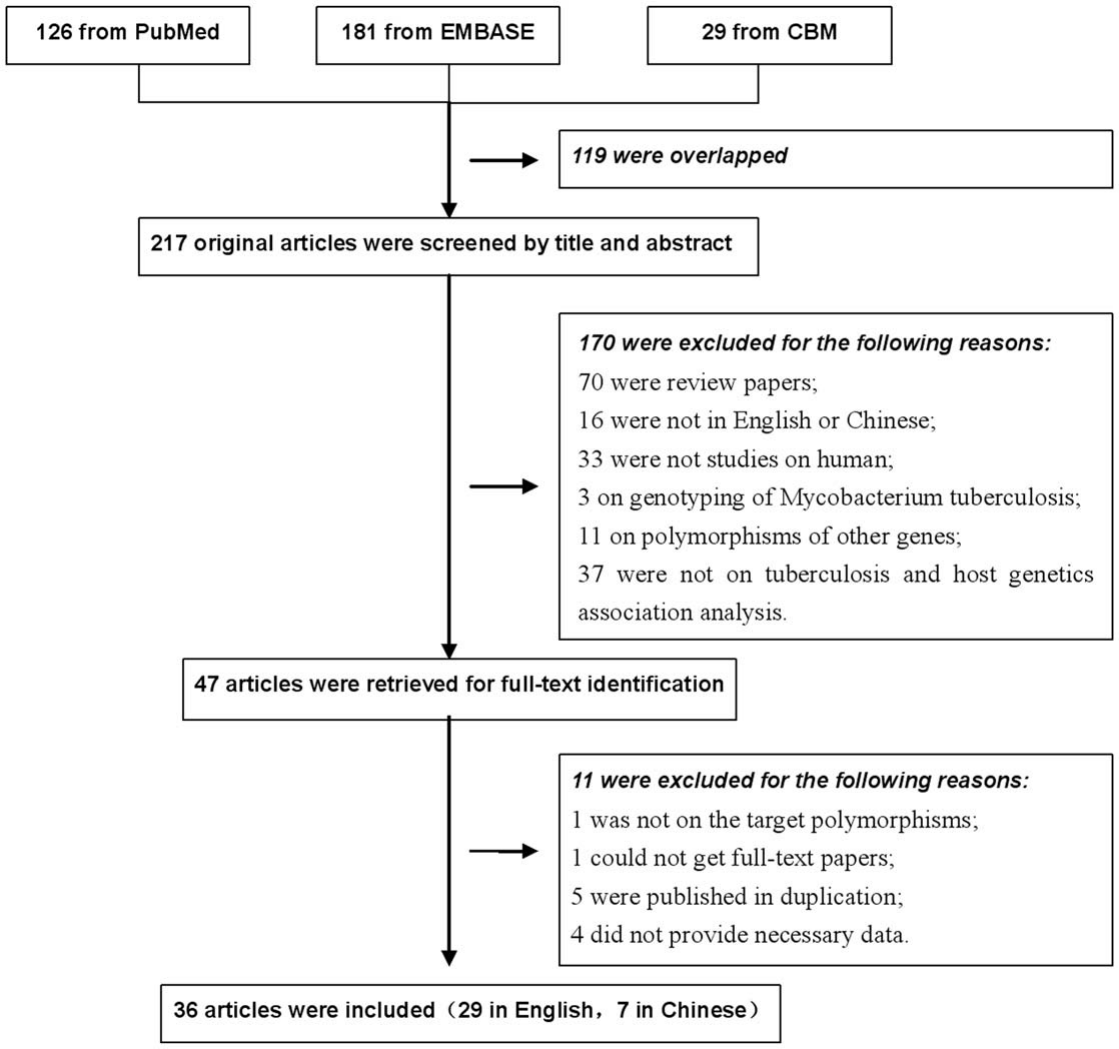
Flow diagram of study identification.

A total of 336 articles were achieved by literature search, from the PubMed, EMBASE and CBM databases, using different combination of key terms. As shown in Figure 1, after excluding those overlapped between the databases, 217 abstracts were retrieved for detailed evaluation. Forty seven studies addressing the association of *SLC11A1* polymorphisms and TB were identified, and full-text article retrieve excluded 11 of them (please refer to Table S1 for more detailed information). Finally, 38 studies from 36 articles, 29 in English [7], [9]–[36]and 7 in Chinese [37]–[43], were included in this review and meta-analysis.

As shown in Table S2 and Table S3, among the included articles, 25 were conduced in Asians and 11 in non-Asians (3 from Africa, 4 from Europeans and 2 from Americans). Thirteen studies and 3 studies specifically addressed pulmonary TB and extra-pulmonary TB, respectively. Seven studies were population based. HIV status of the studied population was considered in 24 studies. There were 13 studies matched controls with cases for major covariates (age, sex, ethnicity, and area of residence). For 3′ UTR, D543N, INT4 and 5′ (GT)n, meta-analyses were conduced within 30, 29, 20 and 12 studies, respectively.

**Figure 2:**
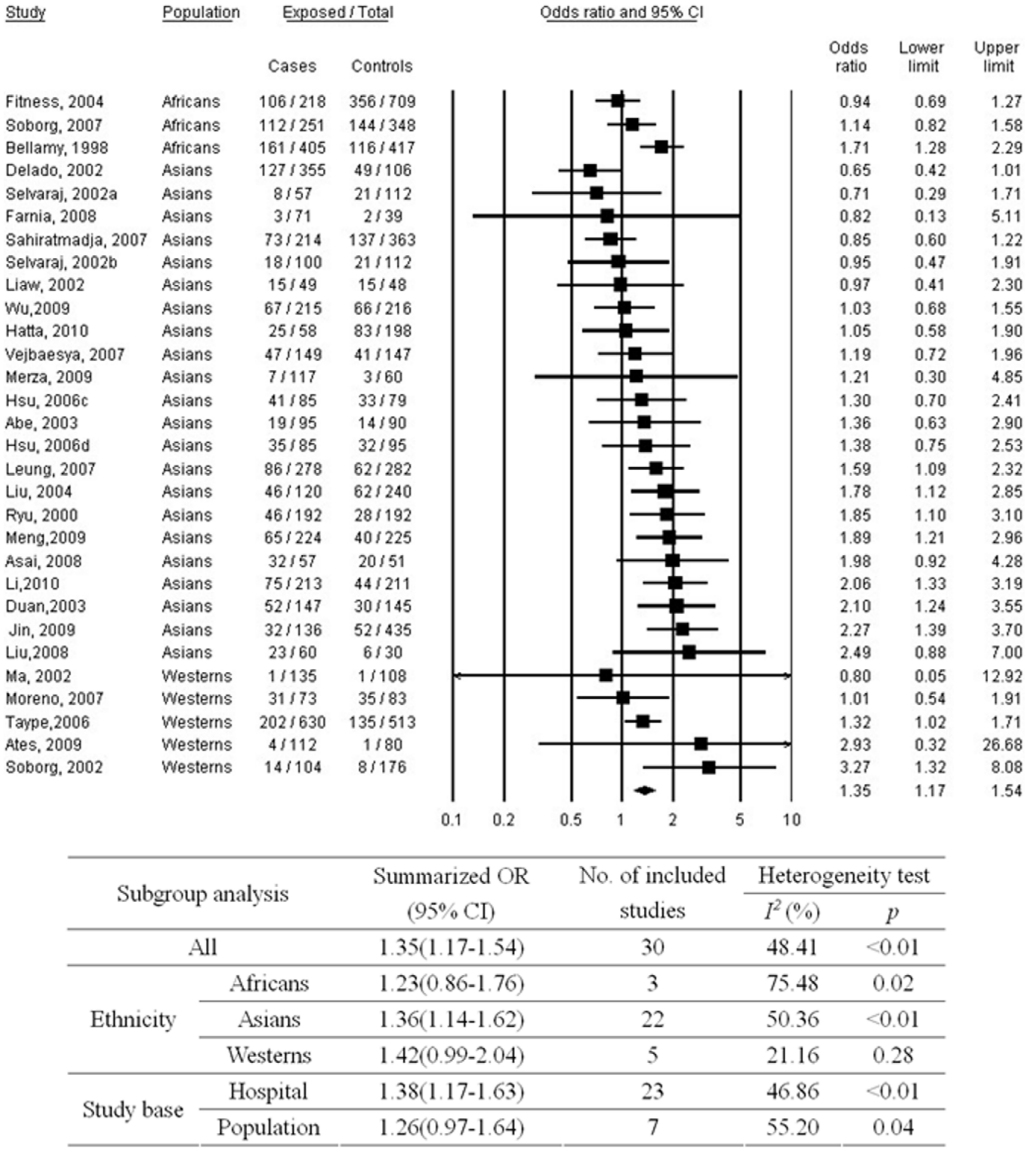
Meta-analysis of the association between tuberculosis and *SLC11A1* 3′ UTR polymorphism (*TGTG- vs. TGTG+/+). a = subgroup of patients with spinal tuberculosis; b = subgroup of patients with pulmonary tuberculosis; c = subgroup of patients who were aboriginal Taiwanese; d = subgroup of patients who were Hans; CI = confidence interval; OR = odds ratio.

Figure 2 shows the associations between *SLC11A1* 3′ UTR polymorphism and TB. Meta-analysis suggested that TGTG- carriage (TGTG−/− and TGTG−/+) might be a risk factor for TB with a summarized OR of 1.35 (95%CI, 1.17–1.54) as compared to TGTG+/+ genotype. Medium heterogeneity between studies (p<0.01; I^2^ = 48.41%) was observed. No evident publication bias was found (p = 0.99 for Begg rank correlation analysis; p = 0.59 for Egger weighted regression analysis). In the stratified analysis, the strength of the association was most evident for Asians, and marginal results were observed for Africans and Westerns. No pronounced difference was found according to different study base.

**Figure 3:**
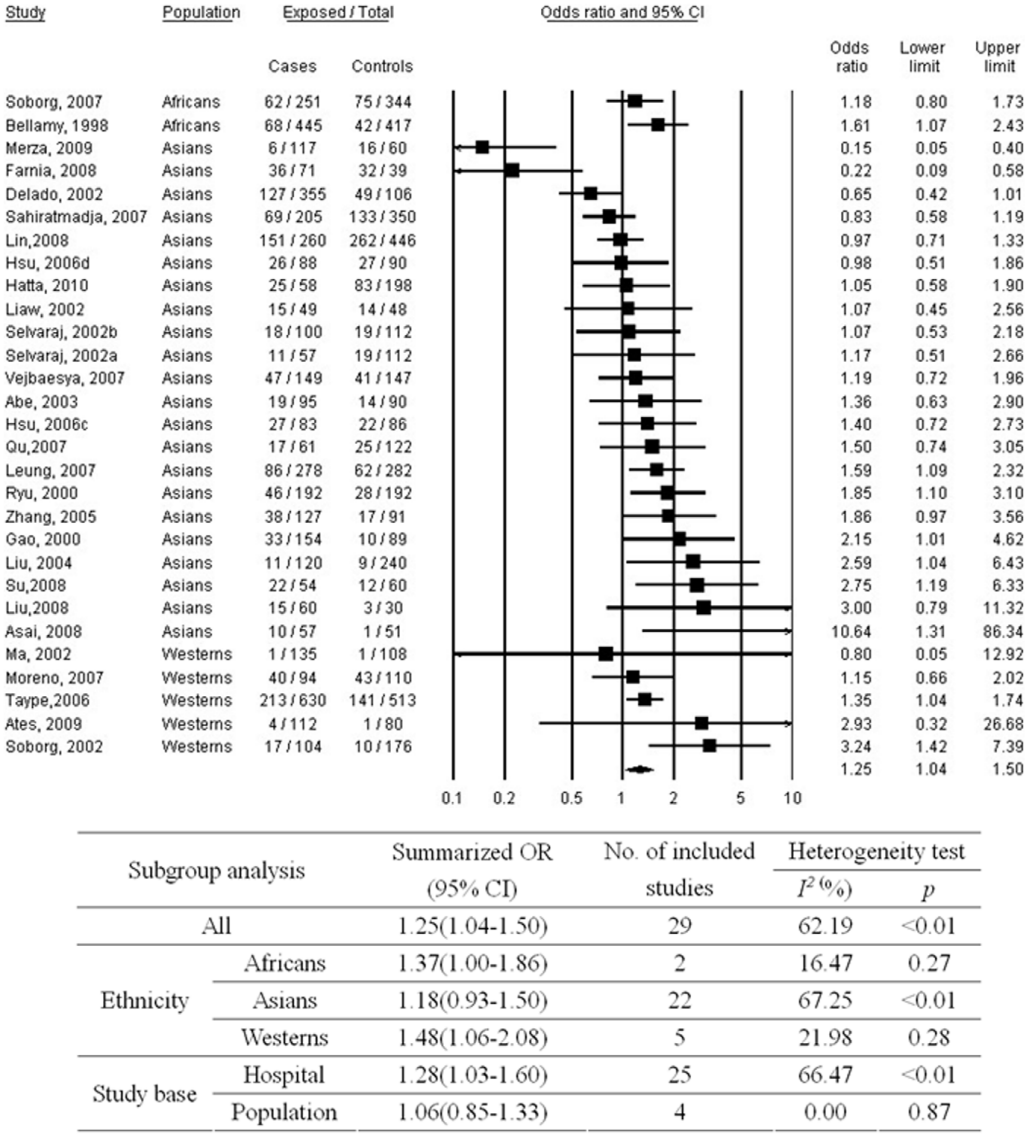
Meta-analysis of the association between tuberculosis and *SLC11A1* D543N polymorphism (*A vs. GG). a = subgroup of patients with spinal tuberculosis; b = subgroup of patients with pulmonary tuberculosis; c = subgroup of patients who were aboriginal Taiwanese; d = subgroup of patients who were Hans; CI = confidence interval; OR = odds ratio.

As shown in Figure 3, good homogeneity was observed between studies addressing D543N polymorphism (p<0.01; I^2^ = 61.26%) and A allele carriage was significantly associated with TB (OR, 1.24; 95% CI, 1.04–1.49). No substantial publication bias was observed (p = 0.39 for Begg rank correlation analysis; p = 0.49 for Egger weighted regression analysis). In the subgroup analyses, significant relations were observed for Africans and Westerns.

**Figure 4:**
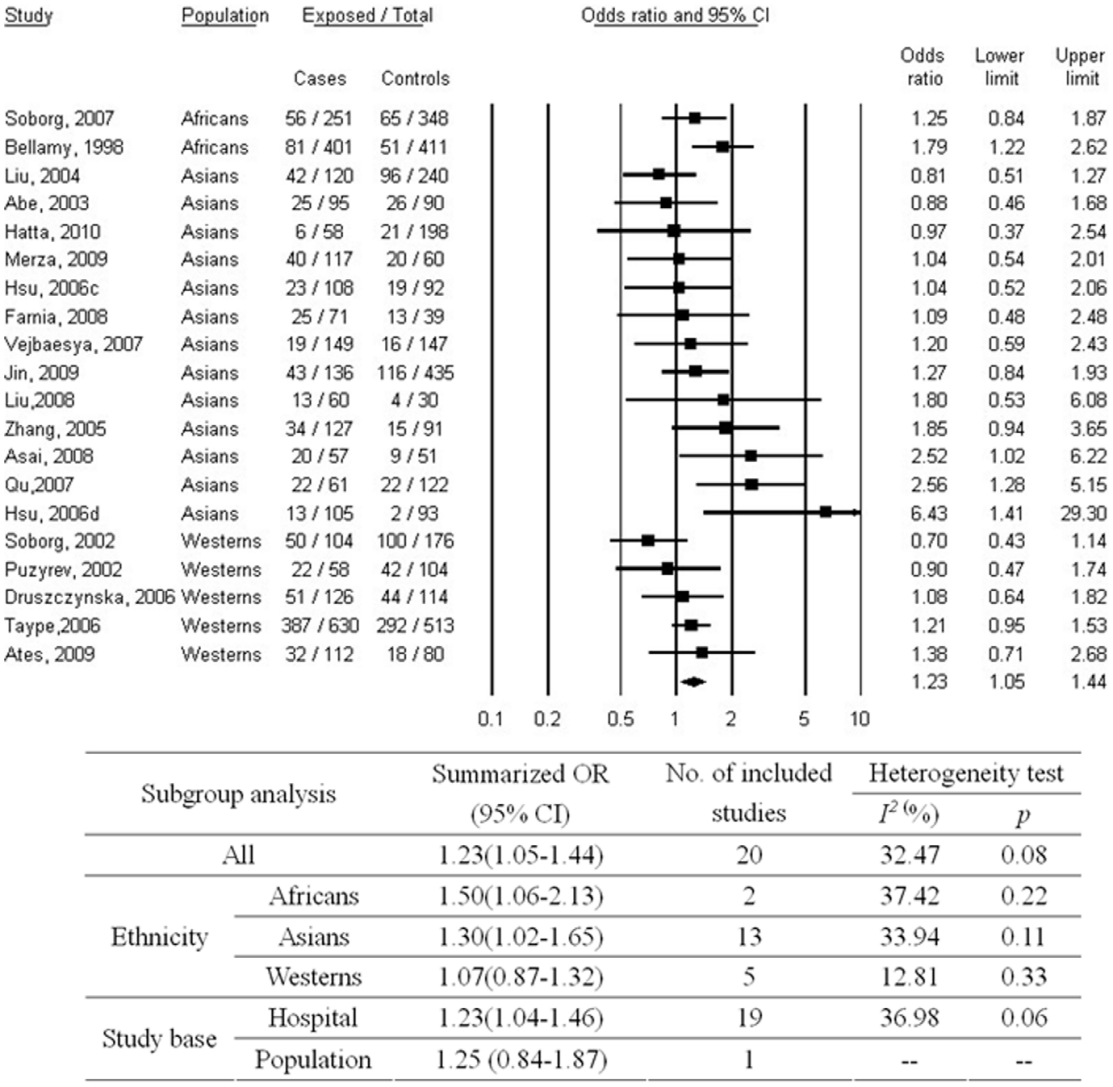
Meta-analysis of the association between tuberculosis and *SLC11A1* INT4 polymorphism (*C vs. GG). c = subgroup of patients who were aboriginal Taiwanese; d = subgroup of patients who were Hans; CI = confidence interval; OR = odds ratio.

As shown in Figure 4, a significant association was found for INT4 C allele carriage (CC+CG) with increased risk of TB as compared to GG genotype (OR, 1.23; 95% CI, 1.04–1.44). Medium heterogeneity between studies (p = 0.08; I^2^ = 32.47%) was observed. No significant publication bias was observed (p = 0.12 for Begg rank correlation analysis; p = 0.35 for Egger weighted regression analysis). The association was not significant any more in Westerns in the subgroup analysis.

**Figure 5:**
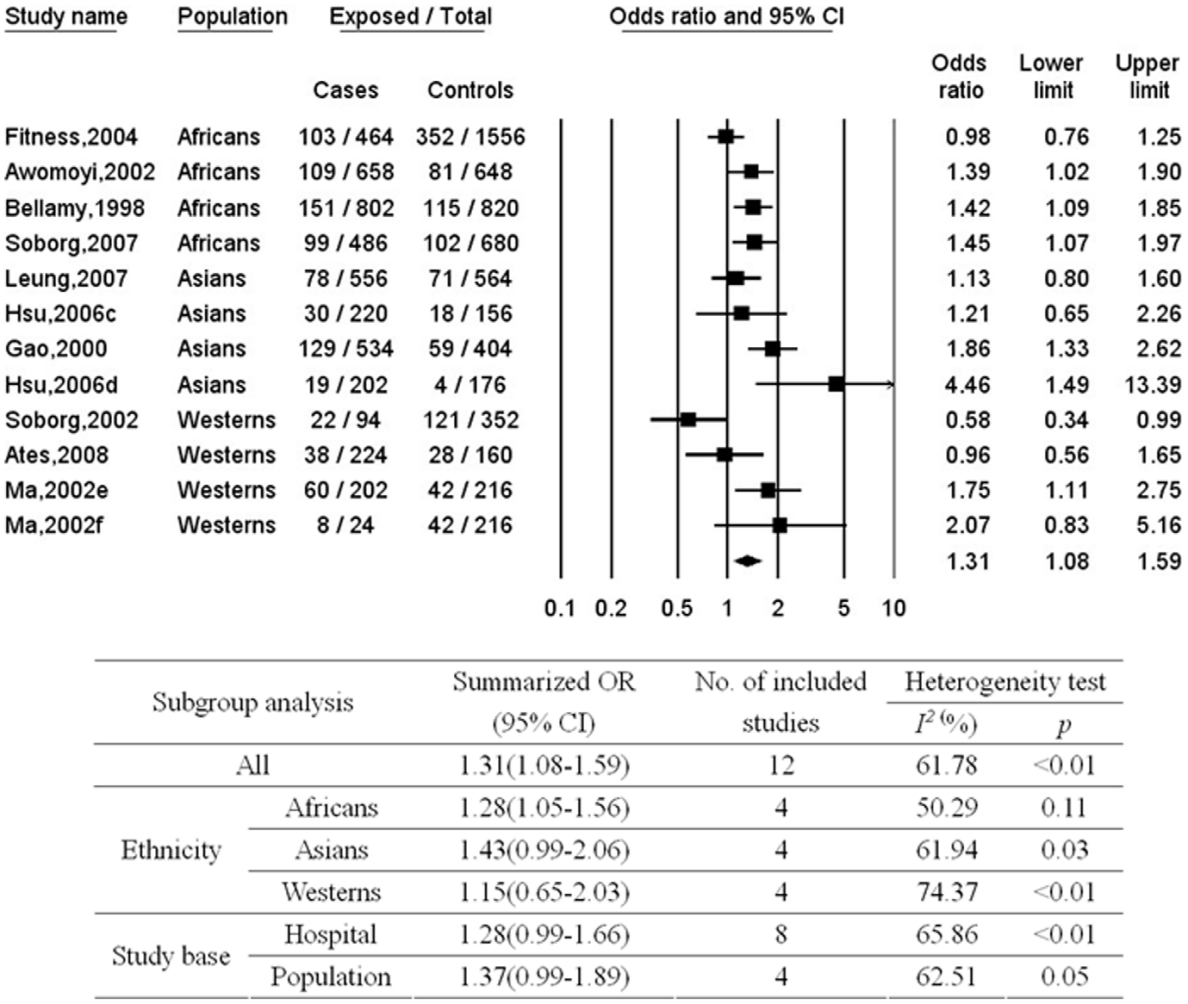
Meta-analysis of the association between tuberculosis and *SLC11A1* 5′ (GT)n polymorphism (allele 3 vs. other alleles). c = subgroup of patients who were aboriginal Taiwanese; d = subgroup of patients who were Hans; e = HIV-negative pulmonary TB patients; f = HIV-negative extra-pulmonary tuberculosis patients; CI = confidence interval; OR = odds ratio.

Meta-analysis of the association between TB and *SLC11A1* 5′ promoter (GT)n polymorphism was shown in Figure 5. As compared to the most commonly distributed allele 3, carriage of other alleles was significantly related to an increased risk of TB with a summarized OR of 1.31 (95%CI, 1.08–1.59). No substantial heterogeneity was observed between studies (p<0.01; I^2^ = 61.78%). No publication bias was found (p = 0.49 for Begg rank correlation analysis; p = 0.51 for Egger weighted regression analysis). In the stratified analyses, similar results were observed between subgroups according to study base, and significant association was found only for Africans.

After excluding studies specifically addressing extra-pulmonary TB, the associations between *SLC11A1* polymorphisms and TB were not substantially changed (see Table S4). Similar associations were observed as well when stratified the analysis on pulmonary TB (see Table S4), which suggests the effect of *SLC11A1* polymorphisms might not be influenced by disease types.

## Discussion

This review addressed the associations between *SLC11A1* polymorphisms and TB susceptibility reported until September 2010. Thirty six articles addressing the most widely studied *SLC11A1* polymorphisms (3′ UTR, D543N, INT4 and 5′ (GT)n) were identified, and their effects were summarized by means of meta-analysis. Significant associations with TB susceptibility were observed for all these four loci. Strength of the associations in the subgroup analyses with respect to study population was not consistent. However, significant association has been observed for all populations (Africans, Asians and Westerns) with at least one of the four loci. Limited number of studies in the stratified analysis and heterogeneity between studies might explain, at least in part, the existing inconsistency.

TB is a serious public health problem in worldwide. Early diagnosis, drug resistance, vaccine and HIV co-infection are major factors influencing efficiency of TB prevention and control [44]. In the past decades, the association between host genetic polymorphisms and TB susceptibility has been widely studied as well. Most of the target loci were localized in genes participating immune response [45]. The NRAMP1 protein is an integral membrane protein expressed exclusively in the lysosomal compartment of monocytes and macrophages [3]. It has been reported that NRAMP1 regulates macrophage activation and is associated with TB susceptibility. Underlying mechanisms have been proposed based on its function as metal transporter [46]. After phagocytosis, NRAMP1 is targeted to the membrane of the microbe-containing phagosome, where it mediates transport of iron and other cations. Iron is essential for biological functions, both for host immune defense and mycobacterial growth. Therefore, NRAMP1 has also been suggested to play a role in determining host susceptibility to other intracellular pathogens and autoimmune diseases [47]. Positive association between *SLC11A1* polymorphisms and these infections and diseases has provided strong evidence for this hypothesis.

In 2006, Li HT and colleagues reported a meta-analysis on *SLC11A1* polymorphisms and TB which based on 14 eligible studies published until December 2004 [8]. In this article, less common alleles at the four most widely studied loci were generally shown to confer an increased risk of TB on their carriers from African and Asian, whereas they were not statistically associated with TB in those of European origin. Authors suggested this ethnic-specific observation may explain in part by Africans and Asians have greater susceptibility to TB than those of Europeans. Meanwhile, authors also mentioned that this hypothesis needed to be proved in future research due to the limited number of included publications and subjects. Our present updated meta-analysis, based on 36 eligible studies until September 2010, did not present an ethic-specific effect of *SLC11A1* polymorphisms. Three ethnic groups were defined with respect to the distribution of study population in the included studies, e.g. Asians, Africans and Westerns (Europeans and Americans). Statistically significant or marginal associations with TB susceptibility were found for the four studied loci in all of the three ethnic groups. Large number of included articles in the present study makes the evidence stronger to propose a consistent effect of *SLC11A1* polymorphisms in different populations. In addition, population-based study has shown to be more powerful for such genetic association analysis. However, only minority of included studies (7 of 36) were conducted as population-based and no substantial different or more pronounced effect was observed as compared to those hospital-based studies.

The stratified analyses suggested different study population and study base might partly explain the moderate heterogeneity between studies observed in our analyses. Further sensitivity analyses were performed as well to explore potential origin of the heterogeneity (see Table S4). When restrict to analyses on pulmonary TB or excluding those studies specifically on extra-pulmonary TB, better homogeneity between studies was observed. Nevertheless, the effects of the polymorphisms were not substantially influenced by such stratified analyses, which suggested that the role of NRAMP1 in the development of TB might not be disease type specific. Further studies addressing the effect of NRAMP1 on pulmonary TB and extra-pulmonary TB, respectively, are warranted to verify this hypothesis.

There are some limitations to this systematic review that should be kept in mind. First, the potential confounding effect of age, sex and ethnicity was not controlled for in more than a half of included studies, which makes the interpretation of the results and stratified analyses difficult. Second, because not all necessary information could be obtained from all included studies, more detailed sub-grouping analysis (e.g. by HIV status or type of TB) could not be performed. Third, the crude division of ethnics groups into ‘Asian’, ‘African’, and ‘Western’ makes the analyses be prone to bias. Further studies from different populations are warranted to verify current findings. Fourth, included studies were restricted to those published in English or Chinese in our study which might introduce potential bias into data analysis as well. Fifth, only 17 included studies mentioned whether their study population was in HWE for the investigated variants [11], [14], [16], [17], [23]–[30], [33]–[35], [39], [43]. Based on the data provided by the articles and own calculations, significant deviations from HWE (p<0.05) in controls were observed for three studies for any of their studied polymorphisms [12], [38], [42]. Their results should be interpreted with more caution. We therefore repeated the meta-analyses after exclusion of these studies. However, this exclusion did not materially affect the results (see Table S4).

In conclusion, this systematic review summarized the associations between *SLC11A1* polymorphisms and TB susceptibility. Our results suggested a consistent association between *SLC11A1* polymorphisms and TB in different populations, which supports the hypothesis that NRAMP1 might play an important role in the host defense to the development of TB. However, due to the moderate strength of the associations, their values to be used for risk prediction should be considered cautiously.

## Materials and Methods

### Literature search

Studies addressing the association between *SLC11A1* polymorphisms and TB were identified by searching for articles in the PubMed, EMBASE and Chinese BioMedical Literature (CBM) Database until 15 September 2010 [5]. Various combinations of the terms “tuberculosis”, “NRAMP1”, “*SLC11A1*”, “polymorphism” and “susceptibility” were used to screen for potentially relevant studies. Additional studies were also identified by means of cross-referencing.

### Inclusion and exclusion criteria

Case-control or cohort studies presenting original data on the associations between *SLC11A1* polymorphisms and TB were included. Only the most widely studied polymorphisms were considered: 3′ UTR (1729+55del4) (rs17235416), D543N (Asp543Asn) (rs17235409), INT4 (469+14G/C) (rs3731865), and 5′ promoter (GT)n (rs34448891). Exclusion criteria were: 1) review articles; 2) studies in languages other than English or Chinese; 2) studies on other polymorphisms other than the target four polymorphisms; 3) previous articles addressing meta-analysis of the associations between *SLC11A1* polymorphisms and TB. If the eligible study was reported in duplication, the article published in English or published earlier was included in this review.

### Data extraction

For all studies, we extracted the following data from original publications: first author and year of publication; distribution of genotypes for each polymorphism among cases and controls; characteristics of the study design and the study population (study base, numbers and mean age of cases and controls, TB diagnosis, HIV status, source of controls, matching criteria and host ethnicity).

### Statistical analysis

Hardy-Weinberg Equilibrium (HWE) was examined in controls by asymptotic Pearson's chi-square test for each polymorphism in each study. The association between polymorphism and TB was estimated by means of odds ratios (OR) and corresponding 95% confidence intervals (CI) comparing cases to controls. Co-dominant model was used for 3′ UTR, D543N, and INT4. The effect of allele 3 carriage was assessed for 5′ (GT)n. Meta-analyses were carried out using Comprehensive Meta-Analysis (V2.0, Biostat, Englewood, NJ, USA). Random effects models were used for meta-analysis, taking into account the possibility of heterogeneity between studies which was tested by the Q test and I^2^ test. Stratified analyses were conducted with respect to study base (hospital or population based) and host ethnicity. The latter was categorized into Africans, Asians and Westerns (Europeans and Americans). Because of the limited number of publications in Americans, they were sub-grouped to Westerns combined with studies from Europeans. Begg rank correlation method and Egger weighted regression method were used to statistically assess publication bias (p<0.05 was considered indicative of statistically significant publication bias). Sensitivity analyses were performed after excluding studies specifically on extra-pulmonary TB, or restricting the analysis specifically on pulmonary TB.

## Supporting Information

Table S1 List of excluded studies and corresponding exclusion criteria.
(DOC)

Table S2 Characteristics of the included studies (Part 1/2).
(DOC)

Table S3 Characteristics of the included studies (Part 2/2).
(DOC)

Table S4 Sensitivity analyses of meta-analyses.
(DOC)

## References

[1] Burgner D, Jamieson SE, Blackwell JM (2006). Genetic susceptibility to infectious diseases: big is beautiful, but will bigger be even better?. Lancet Infect Dis.

[2] Motsinger AA, Haas DW, Hulgan T, Ritchie MD (2007). Human genomic association studies: a primer for the infectious diseases specialist.. J Infect Dis.

[3] Canonne-Hergaux F, Gruenheid S, Govoni G, Gros P (1999). The Nramp1 protein and its role in resistance to infection and macrophage function.. Proc Assoc Am Physicians.

[4] Dye C, Williams BG (2010). The population dynamics and control of tuberculosis.. Science.

[5] Gao L, Tao Y, Zhang L, Jin Q (2010). Vitamin D receptor genetic polymorphisms and tuberculosis: updated systematic review and meta-analysis.. Int J Tuberc Lung Dis.

[6] Pacheco AG, Moraes MO (2009). Genetic polymorphisms of infectious diseases in case-control studies.. Dis Markers.

[7] Bellamy R, Ruwende C, Corrah T, McAdam KP, Whittle HC (1998). Variations in the NRAMP1 gene and susceptibility to tuberculosis in West Africans.. N Engl J Med.

[8] Li HT, Zhang TT, Zhou YQ, Huang QH, Huang J (2006). SLC11A1 (formerly NRAMP1) gene polymorphisms and tuberculosis susceptibility: a meta-analysis.. Int J Tuberc Lung Dis.

[9] Gao PS, Fujishima S, Mao XQ, Remus N, Kanda M (2000). Genetic variants of NRAMP1 and active tuberculosis in Japanese populations. International Tuberculosis Genetics Team.. Clin Genet.

[10] Ryu S, Park YK, Bai GH, Kim SJ, Park SN (2000). 3′UTR polymorphisms in the NRAMP1 gene are associated with susceptibility to tuberculosis in Koreans.. Int J Tuberc Lung Dis.

[11] Awomoyi AA, Marchant A, Howson JMM, McAdam KPWJ, Blackwell JM (2002). Interleukin-10, polymorphism in SLC11A1 (formerly NRAMP1), and susceptibility to tuberculosis.. J Infect Dis.

[12] Delgado JC, Baena A, Thim S, Goldfeld AE (2002). Ethnic-specific genetic associations with pulmonary tuberculosis.. J Infect Dis.

[13] Liaw YS, Tsai-Wu JJ, Wu CH, Hung CC, Lee CN (2002). Variations in the NRAMP1 gene and susceptibility of tuberculosis in Taiwanese.. Int J Tuberc Lung Dis.

[14] Ma X, Dou S, Wright JA, Reich RA, Teeter LD (2002). 5′ dinucleotide repeat polymorphism of NRAMP1 and susceptibility to tuberculosis among Caucasian patients in Houston, Texas.. Int J Tuberc Lung Dis.

[15] Selvaraj; P (2002). NRAMP1 gene polymorphism in pulmonary and spinal tuberculosis.. Curr Sci.

[16] Puzyrev VP, Freidin MB, Rudko AA, Strelis AK, Kolokolova OV (2002). [Polymorphisms of the candidate genes for genetic susceptibility to tuberculosis in the Slavic population of Siberia: a pilot study].. Mol Biol (Mosk).

[17] Soborg C, Andersen AB, Madsen HO, Kok-Jensen A, Skinhoj P (2002). Natural resistance-associated macrophage protein 1 polymorphisms are associated with microscopy-positive tuberculosis.. J Infect Dis.

[18] Abe T, Iinuma Y, Ando M, Yokoyama T, Yamamoto T (2003). NRAMP1 polymorphisms, susceptibility and clinical features of tuberculosis.. J Infect.

[19] Fitness J, Floyd S, Warndorff DK, Sichali L, Malema S (2004). Large-scale candidate gene study of tuberculosis susceptibility in the Karonga district of northern Malawi.. Am J Trop Med Hyg.

[20] Liu W, Cao WC, Zhang CY, Tian L, Wu XM (2004). VDR and NRAMP1 gene polymorphisms in susceptibility to pulmonary tuberculosis among the Chinese Han population: A case-control study.. Int J Tuberc Lung Dis.

[21] Zhang W, Shao L, Weng X, Hu Z, Jin A (2005). Variants of the natural resistance-associated macrophage protein 1 gene (NRAMP1) are associated with severe forms of pulmonary tuberculosis.. Clin Inhect Dis.

[22] Druszczynska M, Strapagiel D, Kwiatkowska S, Kowalewicz-Kulbat M, Rozalska B (2006). Tuberculosis bacilli still posing a threat. Polymorphism of genes regulating anti-mycobacterial properties of macrophages.. Pol J Microbiol.

[23] Hsu YH, Chen CW, Sun HS, Jou R, Lee JJ (2006). Association of NRAMP 1 gene polymorphism with susceptibility to tuberculosis in Taiwanese aboriginals.. J Formos Med Assoc.

[24] Taype CA, Castro JC, Accinelli RA, Herrera-Velit P, Shaw MA (2006). Association between SLC11A1 polymorphisms and susceptibility to different clinical forms of tuberculosis in the Peruvian population.. Infect Genet Evol.

[25] Leung KH, Yip SP, Wong WS, Yiu LS, Chan KK (2007). Sex- and age-dependent association of SLC11A1 polymorphisms with tuberculosis in Chinese: A case control study.. BMC Infect Dis.

[26] Nino-Moreno P, Portales-Perez D, Hernandez-Castro B, Portales-Cervantes L, Flores-Meraz V (2007). P2X7 and NRAMP1/SLC11 A1 gene polymorphisms in Mexican mestizo patients with pulmonary tuberculosis.. Clin Exp Immunol.

[27] Qu Y, Tang Y, Cao D, Wu F, Liu J (2007). Genetic polymorphisms in alveolar macrophage response-related genes, and risk of silicosis and pulmonary tuberculosis in Chinese iron miners.. Int J Hyg Environ Health.

[28] Sahiratmadja E, Wieringa FT, van Crevel R, de Visser AW, Adnan I (2007). Iron deficiency and NRAMP1 polymorphisms (INT4, D543N and 3′UTR) do not contribute to severity of anaemia in tuberculosis in the Indonesian population.. Brit J Nutr.

[29] Soborg C, Andersen AB, Range N, Malenganisho W, Friis H (2007). Influence of candidate susceptibility genes on tuberculosis in a high endemic region.. Mol Immunol.

[30] Vejbaesya S, Chierakul N, Luangtrakool P, Sermduangprateep C (2007). NRAMP1 and TNF-(alpha) polymorphisms and susceptibility to tuberculosis in Thais.. Respirology.

[31] Asai S, Abe Y, Fujino T, Masukawa A, Arami S (2008). Association of the SLC11A1 gene polymorphisms with susceptibility to mycobacterium infections in a japanese population.. Infect Dis Clin Pract.

[32] Farnia P, Pajand O, Anoosheh S, Tabarsi P, Dizaji MK (2008). Comparison of Nramp1 gene polymorphism among TB health care workers and recently infected cases; assessment of host susceptibility.. Tanaffos.

[33] Ates O, Dalyan L, Musellim B, Hatemi G, Turker H (2009). NRAMP1 (SLC11A1) gene polymorphisms that correlate with autoimmune versus infectious disease susceptibility in tuberculosis and rheumatoid arthritis.. Int J Immunogenet.

[34] Jin J, Sun L, Jiao W, Zhao S, Li H (2009). SLC11A1 (Formerly NRAMP1) gene polymorphisms associated with pediatric tuberculosis in China.. Clin Infect Dis.

[35] Merza M, Farnia P, Anoosheh S, Varahram M, Kazampour M (2009). The NRAMPI, VDR and TNF-alpha gene polymorphisms in Iranian tuberculosis patients: the study on host susceptibility.. Braz J Infect Dis.

[36] Hatta M, Ratnawati, Tanaka M, Ito J, Shirakawa T (2010). NRAMP1/SLC11A1 gene polymorphisms and host susceptibility to Mycobacterium tuberculosis and M. leprae in South Sulawesi, Indonesia.. Southeast Asian J Trop Med Public Health.

[37] Duan HF, Zhou XH, Ma Y, Li CY, Chen XY (2003). A Study on The Association of 3′UTR Polymorphisms of NRAM P1 Gene with Susceptibility to Tuberculosis in Hans.. Tuber&Thor tumor.

[38] Lin R, Bo JP, Cai WW, Li X, Lin F (2008). Study on relationship between DNA polymorphisms of natural resistance associated macrophage proteinl gene and susceptibility to tuberculosis in Hainan Li ethnic group.. J Clin Med in Practic.

[39] Liu ZB, Xiao HP, Sha W, Zheng RJ, Liu YD (2008). A case-control study on distribution of SLCllA1 gene polymorphisms between new and recrudescent cases of pulmonary tuberculosis.. J Tongji Uni (Med Sci).

[40] Su BH, Wang ZL, Li SJ, Xiao B (2008). A study on the association of D543N polymorphisms of NRAM P1 gene with susceptibility to tuberculosisin in Han ethnic.. Ningxia Med.

[41] Meng XJ, Wu FT, Yan D, Wang X, Li CZ (2009). Study on the association of 3′UTR polymorphisms of NRAMPl gene with susceptibility to tuberculosis in Uighurs.. Chin J Microbiol Immunol.

[42] Wu JD, Li CZ, Meng XJ, Liu JM, Wu F (2009). A study on the association of 3 UTR polymorphisms of NRAM P1 gene with susceptibility to tuberculosis in Hans.. J Nongkan Med.

[43] Li CZ, Jia XB, Meng XJ, Zhang WJ (2010). Research on the Association of 3′UTR Polymorphisms of NRAMP1 Gene with Susceptibility to Tuberculosis in Hazakhs of Xinjiang.. Life Sci Res.

[44] Abu-Raddad LJ, Sabatelli L, Achterberg JT, Sugimoto JD, Longini IM (2009). Epidemiological benefits of more-effective tuberculosis vaccines, drugs, and diagnostics.. Proc Natl Acad Sci U S A.

[45] Yim JJ, Selvaraj P Genetic susceptibility in tuberculosis.. Respirology.

[46] McDermid JM, Prentice AM (2006). Iron and infection: effects of host iron status and the iron-regulatory genes haptoglobin and NRAMP1 (SLC11A1) on host-pathogen interactions in tuberculosis and HIV.. Clin Sci (Lond).

[47] Blackwell JM, Goswami T, Evans CA, Sibthorpe D, Papo N (2001). SLC11A1 (formerly NRAMP1) and disease resistance.. Cell Microbiol.

