# Supplementary material for: SLC11A1 (NRAMP1) Polymorphisms and Tuberculosis Susceptibility: Updated Systematic Review and Meta-Analysis
Source: PLoS One. 2011 Jan 25;6(1):e15831. doi: 10.1371/journal.pone.0015831 (PMC3026788; doi:10.1371/journal.pone.0015831)
Supplement: Table S1 — List of excluded studies and corresponding exclusion criteria. (DOC) [file pone.0015831.s001.doc]

**Table S1. List of excluded studies and corresponding exclusion criteria**

| **Exclusion criteria** | **Articles** |
| --- | --- |
| Not addressing widely studied target polymorphisms | 1. Awomoyi A, Sirugo G, Newport MJ, Tishkoff S (2006) Global distribution of a novel trinucleotide microsatellite polymorphism (ATA)n in intron 8 of the SLC11A1 gene and susceptibility to pulmonary tuberculosis. Int J Immunogenet 33: 11-15. |
| Could not get full-text paper even corresponded with authors | 1. Stagas MK, Orfanidou D, Kostopoulos C, Syriou S, Rezcko M, et al. (2010) Incidence and distribution of the polymorphisms in the NRAMP1 (SLC11a1) gene and susceptibility to tuberculosis in Greek population. Review of Clinical Pharmacology and Pharmacokinetics, International Edition 24: 214-216. |
| Needed data could not be extracted from the article | 1. AN Yc, Feng FM, Yuan JX, Ji CM, Wang YH, et al. (2006) Study on the association of INT4 and 3' UTR polymorphism of natural-resistance-associated macrophage protein 1 gene with susceptibility to pulmonary tuberculosis. Chin J Epid 27: 37-41. 2. Hoal EG, Lewis LA, Jamieson SE, Tanzer F, Rossouw M, et al. (2004) SLC11A1 (NRAMP1) but not SLC11A2 (NRAMP2) polymorphisms are associated with susceptibility to tuberculosis in a high-incidence community in South Africa. International Journal of Tuberculosis and Lung Disease 8: 1464-1471. 3. Ji CM, An YC, Li J, Wang YH (2006) A study on the association of D543N and 3'UTR polymorphism of NRAMP1 gene with susceptibility to pulmonary tuberculosis. New Medical Science 5: 1-4. 4. Velez DR, Hulme WF, Myers JL, Stryjewski ME, Abbate E, et al. (2009) Association of SLC11A1 with tuberculosis and interactions with NOS2A and TLR2 in African-Americans and Caucasians. Int J Tuberc Lung Dis 13: 1068-1076. |
| Published in duplication | 1. Duan HF, Zhou XH, Ma Y, Li CY, Chen XY, et al. (2003) A Study on The Association of 3'UTR Polymorphisms of NRAM P1 Gene with Susceptibility to Tuberculosis in Hans. Chin J Tubem Respir Dis 15: 6. 2. Liu W, ZHANG CY, Tian L, Li CZ, Wu XM, et al. (2003) A case·control study on VDR and NRAMP1 gene polymorphisms with susceptibility to pulmonary tuberculsis in Chinese Han Population. Bull Acad Mil Med Sci 27: 409-412. 3. Liu W, ZHANG CY, Tian L, Li CZ, Wu XM, et al. (2003) A case-control study on natural-resistance-associated macrophage protein 1 gene polymorphisms and susceptibility to pulmonary tuberculosis. Chin J Prev Med 37: 408-411. 4. Qu YB, Tang YX, Zhang ZB, Zhu R, Liu J, et al. (2006) Relationship between single nucleotide polymorphisms of NRAMP1 gene and susceptibility to pulmonary tuberculosis in workers exposed to silica dusts. Chin J Ind Hyg Occup Dis 24: 531-533. 5. Shao LY, Weng XH, Hu ZY, Jin AJ, Zhang WH (2004) The relationship between polymorphisms of NRAMP1 gene and susceptibility to tuberculosis in Chinese Han nation. Chin J Infect Dis 22: 302-305. |
