# Supplementary material for: SLC11A1 (NRAMP1) Polymorphisms and Tuberculosis Susceptibility: Updated Systematic Review and Meta-Analysis
Source: PLoS One. 2011 Jan 25;6(1):e15831. doi: 10.1371/journal.pone.0015831 (PMC3026788; doi:10.1371/journal.pone.0015831)
Supplement: Table S2 — Characteristics of the included studies (Part 1/2). (DOC) [file pone.0015831.s002.doc]

**Table S2. Characteristics** of the included studies (Part 1/2).

| **First author, publish year** | **Population** | **No. of Pat/Cont** | **Study base** | **Proportion of males in Pat/Cont (%)** | **Mean age (SD)** | | **Diagnosis of TB^§^** | **Source of controls** | **HIV status** | **Matching criteria** |
| --- | --- | --- | --- | --- | --- | --- | --- | --- | --- | --- |
|  |  |  |  |  | **Pat** | **cont** |  |  |  |  |
| Hatta, 2010 | Indonesian | 58/198 | Hospital | 50.0/48.5 | 34.0 (13.1) | 32.0 (12.9) | B | Blood donors | NA | NA |
| Li, 2010 | Chinese | 213/211 | Population | 44.6/42.7 | 27.0 (10.1) | 29.0 (9.0) | E | Healthy adults | Negatives | NA |
| Ates, 2009 | Pakistani | 112/80 | Hospital | 62.5/60.0 | 46.3 (11.4) | 53.1 (7.3) | F | Healthy individuals | NA | NA |
| Jin, 2009 | Chinese | 136/435 | Hospital | 58.8/59.1 | 5.7 (4.6) | 5.8 (4.1) | E | Pediatric surgical patients | Negatives | Age, sex, ethnicity |
| Meng, 2009 | Chinese | 224/225 | Population | 50.4/50.2 | 44.0 | 49.0 | D | Healthy adults | Negatives | NA |
| Merza, 2009 | Iranian | 117/60 | Hospital | NA | NA | NA | D | Healthy adults | NA | Age, sex, ethnicity |
| Wu, 2009 | Chinese | 215/216 | Population | 50.2/51.4 | 42.0 | 40.0 | E | Healthy adults | Negatives | NA |
| Asai, 2008 | Japanese | 57/51 | Hospital | 75.4/58.8 | 48.0 | 51.0 | E | Healthy adults | Negatives | Ethnicity |
| Farnia, 2008 | Iranian | 71/39 | Hospital | 45.0/48.0 | 46.7 | 34.1 | A | Staff of TB clinic | Negatives | NA |
| Lin, 2008 | Chinese | 130/233 | Population | 73.1/43.3 | 47.0 | 49.0 | C | Healthy adults | Negatives | NA |
| Liu, 2008 | Chinese | 60/30 | Hospital | 66.6/66.6 | NA | 37.3 (5.7) | D | Healthy adults | Negatives | NA |
| Su, 2008 | Chinese | 54^#^/60 | Hospital | 55.6/53.3 | 46.0 | 40.0 | H | Healthy adults | Negatives | NA |
| Leung, 2007 | Chinese | 278*/282 | Hospital | 74.0/74.0 | 65.0 (18.4) | 65.0 (18.2) | B | TB-free patients and blood donors | NA | Age, sex |
| Moreno, 2007 | Mexican | 94*/100 | Population | 51.1/NA | NA | NA | E | TB-free individuals | NA | NA |
| Qu, 2007 | Chinese | 61*/122 | Hospital | 100/100 | NA | NA | D | PTB-free Miners | Negatives | Age, sex |
| Sahiratmadja, 2007 | Indonesian | 378*/436 | Hospital | 59.5/56.2 | 29.0 | 33.0 | D | Healthy individuals | Mixed | Age, sex |
| Soborg, 2007 | Tanzanian | 399*/408 | Population | NA | NA | NA | B | Healthy individuals | Negatives | NA |
| Vejbaesya, 2007 | Thais | 149/147 | Hospital | 58.4/58.5 | NA | NA | E | Blood donors | NA | NA |
| Druszczy, 2006 | Polish | 126/124 | Hospital | NA | 51.0 (16.0) | 50.0 (14.0) | B | TB-free volunteers | NA | NA |
| Hsu, 2006 | Chinese | 105/95 | Hospital | 51.3/41.7 | 50.1 (22.9) | 45.9 (13.7) | F | Blood donors | NA | Ethnicity |
| Hsu, 2006 | Chinese | 110/92 | Hospital | 55.2/42.9 | 35.1 (22.2) | 48.3 (13.1) | F | Blood donors | NA | Ethnicity |
| Taype, 2006 | Peruvian | 507/513 | Hospital | 100/100 | 29.0 (11.4) | 32.6 (9.4) | F | Adult male donors | Negatives | Area, ethnicity |
