# Supplementary material for: SLC11A1 (NRAMP1) Polymorphisms and Tuberculosis Susceptibility: Updated Systematic Review and Meta-Analysis
Source: PLoS One. 2011 Jan 25;6(1):e15831. doi: 10.1371/journal.pone.0015831 (PMC3026788; doi:10.1371/journal.pone.0015831)
Supplement: Table S3 — Characteristics of the included studies (Part 2/2). (DOC) [file pone.0015831.s003.doc]

**Table S3. Characteristics of the included studies (Part 2**/2).

| Zhang, 2005 | Chinese | 127*/91 | Hospital | 70.9/65.9 | 52.3 (13.3) | NA | D | Blood donors | Negatives | NA |
| --- | --- | --- | --- | --- | --- | --- | --- | --- | --- | --- |
| Fitness, 2004 | Malawi | 514/913 | Population | NA | NA | NA | D | Healthy adults | Negatives | Age, sex, area of residence |
| Liu, 2004 | Chinese | 120*/240 | Hospital | 100/100 | 27.7 (12.7) | 27.3 (9.2) | E | Unrelated male servicemen | Negatives | NA |
| Abe, 2003 | Japanese | 95/90 | Hospital | 73.7/56.7 | 58.0 | 61.4 | B | Healthy adults | Negatives | Ethnic, area of residence |
| Duan, 2003 | Chinese | 147/145 | Hospital | 52.4/49.7 | 43.5 | 48.9 | D | Healthy adults | Negatives | NA |
| Awomoyi, 2002 | Gambian | 329*/324 | Hospital | 100/100 | 36.0 | 32.0 | C | Blood donors | Negatives | Age, ethnicity |
| Delado, 2002 | Cambodian | 358/106 | Hospital | 37.3/43.9 | 42.2 (14.1) | 37.5 (12.9) | F | Healthy adults | Negatives | NA |
| Liaw, 2002 | Chinese | 48/49 | Hospital | 75.5/64.5 | 56.0 | 49.0 | B | Healthy adults | Negatives | NA |
| Ma, 2002 | American | 135/108 | Hospital | 76.1/37.0 | 52.2 (11.2) | 61.4 (14.8) | B | TB-free clinic patients | Negatives | NA |
| Puzyrev, 2002 | Russian | 58/127 | Hospital | NA | 19.3 (15.2) | 39.1 (3.3) | NA | Pat’s spouses and staff of clinic | NA | Ethnicity |
| Selvaraj, 2002 | Indian | 57^&^/112 | Hospital | 50.1/47.3 | 39.5 (3.3^†^)  45.8 (2.5^‡^) | 40.5 (1.4^†^)  36.8 (1.1^‡^) | G | Pat’s family member, colleague | NA | Ethnicity |
| Selvaraj, 2002 | Indian | 100*/112 | Hospital | 77.0/47.3 | 40.5 (1.3^†^)  38.9 (2.5^‡^) | 40.5 (1.4^†^)  36.8 (1.1^‡^) | B | Pat’s family member, colleague | NA | Ethnicity |
| Gao,2000 | Japanese | 202*/267 | Hospital | 86.1/69.3 | 57.8 (15.8) | 45.4 (16.8) | A | Health check-up | Negatives | NA |
| Ryu, 2000 | Korean | 192/192 | Hospital | NA | NA | NA | D | Health check-up | NA | NA |
| Bellamy, 1998 | Gambian | 410*/417 | Hospital | 67.4/100 | 34.7 (13.2) | 30.3 (7.5) | A | Blood donors | Negatives | Ethnicity |

Abbreviation: NA, not available; Cont, control; Pat, patient; PTB, pulmonary tuberculosis; SD, standard deviation; TB, tuberculosis.

^*^ Cases with pulmonary TB.

^&^ Cases with extra-pulmonary tuberculosis.

^#^ Cases with osseous tuberculosis.

^§^Diagnosis of tuberculosis in the different study was subgrouped into the following categories: A. sputum smear microscopy; B. sputum smear microscopy, sputum culture; C. sputum smear microscopy, X-ray. D. sputum smear microscopy, sputum culture, X-ray; E. sputum smear microscopy, sputum culture, X-ray, symptom; F. sputum smear microscopy, sputum culture, X-ray, symptom, pathology; G. X-ray, symptom; H. symptom, radiology, pathology.
