# Supplementary material for: SLC11A1 (NRAMP1) Polymorphisms and Tuberculosis Susceptibility: Updated Systematic Review and Meta-Analysis
Source: PLoS One. 2011 Jan 25;6(1):e15831. doi: 10.1371/journal.pone.0015831 (PMC3026788; doi:10.1371/journal.pone.0015831)
Supplement: Table S4 — Sensitivity analyses of meta-analyses. (DOC) [file pone.0015831.s004.doc]

**Table S4. Sensitivity analyses of meta-analyses**

| **Subgroup analysis** | | **Summarized odds ratio (95% CI)** | **No. of included studies** | ***I^2^*(%)** | ***p*** |
| --- | --- | --- | --- | --- | --- |
| 3’ UTR | All | 1.35(1.17-1.54) | 30 | 48.41 | <0.01 |
|  | Studies specifically on pulmonary TB | 1.24(0.98-1.56) | 8 | 54.65 | 0.03 |
|  | Studies specifically on extra-pulmonary TB were excluded | 1.36(1.19-1.56) | 29 | 48.42 | <0.01 |
|  | Study with controls not in HWE excluded | 1.41(1.24-1.61) | 28 | 38.29 | 0.02 |
| D543N | All | 1.25(1.04-1.50) | 29 | 62.19 | <0.01 |
|  | Studies specifically on pulmonary TB | 1.35(1.11-1.64) | 11 | 28.99 | 0.17 |
|  | Studies specifically on extra-pulmonary TB were excluded | 1.22(1.01-1.48) | 27 | 63.01 | <0.01 |
|  | Study with controls not in HWE excluded | 1.31(1.08-1.59) | 27 | 58.59 | <0.01 |
| INT4 | All | 1.23(1.05-1.44) | 20 | 32.47 | 0.08 |
|  | Studies specifically on pulmonary TB | 1.47(1.01-2.12) | 5 | 63.71 | 0.03 |
|  | Studies specifically on extra-pulmonary TB were excluded | 1.23(1.05-1.44) | 20 | 32.47 | 0.08 |
|  | Study with controls not in HWE excluded | 1.23(1.05-1.44) | 20 | 32.47 | 0.08 |
| 5’ (GT)n | All | 1.31(1.08-1.59) | 12 | 61.78 | <0.01 |
|  | Studies specifically on pulmonary TB | 1.43(1.25-1.65) | 5 | 2.66 | 0.39 |
|  | Studies specifically on extra-pulmonary TB were excluded | 1.29(1.06-1.57) | 11 | 63.94 | <0.01 |
|  | Study with controls not in HWE excluded | 1.31(1.08-1.59) | 12 | 61.78 | <0.01 |

Abbreviation: CI, confidence interval; OR, odds ratio.
